# Interrogation of Phenotypic Plasticity between Epithelial and Mesenchymal States in Breast Cancer

**DOI:** 10.3390/jcm8060893

**Published:** 2019-06-21

**Authors:** Sugandha Bhatia, James Monkman, Tony Blick, Cletus Pinto, Mark Waltham, Shivashankar H Nagaraj, Erik W Thompson

**Affiliations:** 1 Institute of Health and Biomedical Innovation, Queensland University of Technology, Brisbane, QLD 4059, Australia; james.monkman@qut.edu.au (J.M.); blick_tony@yahoo.com.au (T.B.); shiv.nagaraj@qut.edu.au (S.H.N.); 2 School of Biomedical Sciences, Faculty of Health, Queensland University of Technology, Brisbane, QLD 4000, Australia; 3 Translational Research Institute, Brisbane, QLD 4102, Australia; 4 Invasion and Metastasis Unit, St. Vincent’s Institute, Melbourne, VIC 3065, Australia; cletusp136@gmail.com (C.P.); mwaltham@unimelb.edu.au (M.W.); 5 Department of Surgery, University of Melbourne, St. Vincent’s Hospital, Melbourne, VIC 3065, Australia

**Keywords:** copy number variations (CNV), epithelial-mesenchymal transition (EMT), intratumoral heterogeneity, mesenchymal-epithelial transition (MET), phenotypic plasticity, single cell-derived clones, whole exome sequencing

## Abstract

Dynamic interconversions between transitional epithelial and mesenchymal states underpin the epithelial mesenchymal plasticity (EMP) seen in some carcinoma cell systems. We have delineated epithelial and mesenchymal subpopulations existing within the PMC42-LA breast cancer cell line by their EpCAM expression. These purified but phenotypically plastic states, EpCAM^High^ (epithelial) and EpCAM^Low^ (mesenchymal), have the ability to regain the phenotypic equilibrium of the parental population (i.e., 80% epithelial and 20% mesenchymal) over time, although the rate of reversion in the mesenchymal direction (epithelial-mesenchymal transition; EMT) is higher than that in the epithelial direction (mesenchymal-epithelial transition; MET). Single-cell clonal propagation was implemented to delineate the molecular and cellular features of this intrinsic heterogeneity with respect to EMP flux. The dynamics of the phenotypic proportions of epithelial and mesenchymal states in single-cell generated clones revealed clonal diversity and intrinsic plasticity. Single cell-derived clonal progenies displayed differences in their functional attributes of proliferation, stemness marker (CD44/CD24), migration, invasion and chemo-sensitivity. Interrogation of genomic copy number variations (CNV) with whole exome sequencing (WES) in the context of chromosome count from metaphase spread indicated that chromosomal instability was not influential in driving intrinsic phenotypic plasticity. Overall, these findings reveal the stochastic nature of both the epithelial and mesenchymal subpopulations, and the single cell-derived clones for differential functional attributes.

## 1. Introduction

Cellular heterogeneity within and among cancers is the subject of considerable research, with evidence of genetic and phenotypic heterogeneity in both normal and neoplastic cells across different tissue types [1,2,3,4]. The proportion of cancer cells in distinct states is often correlated with tumor type and grade [5,6,7,8,9]. The degree of heterogeneity (whether inter-tumoral or intra-tumoral) is also considered as a significant predictor of metastatic potential [10,11,12]. In breast cancer, molecular profiling of patient tumors led to the identification of transcriptional breast cancer subtypes, categorized as Basal, Luminal A, Luminal B, Her2+, Claudin-low and Normal-like [13,14,15,16], with further sub-classification of the triple negative breast cancers (TNBC) into 10 distinct groups [17]. Cancer cells in these differing phenotypic states exhibit important differences in their functional properties and clinical course [18,19,20]. 

Cellular plasticity allowing lineage transition is generally silenced in adult tissues except in undifferentiated stem cells [21]. Epithelial mesenchymal plasticity (EMP) is not restricted to transition across binary epithelial and mesenchymal states. In fact, cancer cell plasticity can be described as the continuum that exists between the forward process, epithelial-mesenchymal transition (EMT), as well as the reverse process, mesenchymal-epithelial transition (MET; reviewed in [22,23]). The activation of plasticity programmes in cancers arises as a pathological consequence of genetic and epigenetic changes in the tumor cells, and/or in response to exogenous stimuli including inflammation, hypoxia, or paracrine signaling ligands, such as transforming growth factor-β (TGF-β) and epidermal growth factor (EGF), that are primarily secreted by the tumor-associated stroma. Within individual tumors, carcinoma cells often exhibit a spectrum of phenotypic states along the EMP axis, or can often adopt a hybrid epithelial/mesenchymal (E/M) phenotype [22,24,25]. 

EMP-specific cellular phenotypes can be isolated using EpCAM, Integrin-β4 or CD44/CD24 expression in basal-like cell lines representing TNBC [26,27,28,29], or by using E-cadherin in mammary carcinoma in mouse PyMT models [30]. Similar work has also shown that basal, luminal and stem-like cancer cell subpopulations, isolated from different breast cancer cell lines, can stably retain intra-tumoral heterogeneity, and that all three populations of cells are able to initiate tumor formation in vivo [29]. The different pathological subtypes of breast and oral cancer cells have also been observed to transition between these states; non-cancer stem cells (CSCs) in the tumor tissue can spontaneously undergo EMT and dedifferentiate into new CSCs, thereby gaining tumorigenic potential [28,29,31,32]. Therefore, this plasticity has the capability to alter the whole cancer landscape, attenuate the oncogenic signaling networks, lead to acquisition of anti-apoptotic features, defend against chemotherapeutics, and reprogram angiogenic and immune cell functions [31,33,34,35,36]. 

Phenotypic diversity in cancer, attributed to both genetic and non-genetic dysregulation, also obscures many of the fundamentally important facets of cancer. Publicly-available cancer datasets, such as TCGA, Geo, ICGC and other resources, carry data obtained from high-throughput transcriptomic analyses, such as microarray, and RNA sequencing performed on whole cancer tissue biopsies. This provides population averages of gene expression levels, which limits its use for quantitatively investigating changes within the heterogeneous cellular subpopulations, highlighting the paramount importance of single cell analysis in these studies. 

Studies have been performed at the single-cell level to evaluate gene-expression and genomic sequencing of distinct cell populations present within varying neoplasms in the breast, liver, kidney, and colon [37,38,39,40], allowing insight into the dynamics of clonal evolution in cancers [41]. The divergent modes of cancer spread were deduced through whole genome and single-nucleus sequencing of 68 samples from 7 high-grade serous ovarian cancers to infer the phylogenetic clades of the purified clones [42]. Population-wide, barcoded, single-cell RNA-sequencing data are emerging and herald a major refinement of our understanding of heterogeneity and plasticity [43,44,45]. Further studies are ongoing to investigate different cancer subtypes at the single-cell level. Variation in phenotypic plasticity within sub-clones has also been studied in breast cancer cell lines utilizing DNA barcode labeling [46], as well as in primary glioblastoma through estimation of copy-number variation of single cells obtained from single-cell RNA sequencing [47]. Dynamics of single cell transitions were also studied in breast cancer cells subjected to paclitaxel treatment to discern specific transcriptional variants responsible for the cell survival, as well as for the ability of cells to recover to their original state [48].

We have employed the PMC42-LA breast cancer cell model, an epithelial subline derived from its mesenchymal parental line, PMC42-ET [49,50,51,52]. The phenotypic heterogeneity that exists along the epithelial–mesenchymal axis was examined and validated in vitro, as well as in a mouse xenograft model. We performed clonal propagation of single cells and interrogated the phenotypically distinct clonal progenies for differential facets of plasticity along the EMP axis in a number of assays. We investigated whether the intrinsic plasticity observed is due to genomic/chromosomal instability through whole exome sequencing of sorted epithelial and mesenchymal states in PMC42-LA. Understanding the cellular dynamics of phenotypic states and how they transition within carcinomas is of particular significance in tumor pathobiology and could provide insights into the predictions of clinical outcomes, such as response to therapies and patient survival. 

## 2. Materials and Methods

### 2.1. Cell Lines and Cell Culture

PMC42-ET (ET) cells were derived from a breast cancer pleural effusion by Dr. Robert Whitehead, Ludwig Institute for Cancer Research, Melbourne, Australia, with appropriate institutional ethics clearance (Institutional review board of the Peter MacCallum Hospital, Melbourne) and patient consent [53,54,55]. The PMC42-LA (LA) subline was derived further from the parental PMC42-ET cells by Dr. Leigh Ackland, Deakin University, Melbourne, Australia, [49,53,54,55] and was found to have more epithelial features than the parental PMC42-ET [51,56]. 

PMC42 cell lines were maintained in Dulbecco’s modified Eagle’s medium (DMEM) containing glucose (4.5 g/L), L-Glutamine (0.5 g/L) and sodium pyruvate (0.1 g/L) (Corning, Catalog number—10-013-CVR), and supplemented with 10% fetal bovine serum (FBS; Gibco^TM^, Thermo, Victoria, Australia) and antibiotics, penicillin and streptomycin (Gibco^TM^, Life Technologies Catalog number—15140122). Cell number and viability was determined by 0.4% trypan blue dye exclusion and loaded onto the TC20^TM^ Automated Cell counter (Bio-Rad). Cells were routinely confirmed negative for *Mycoplasma* (MycoAlert^TM^ mycoplasma detection kit, Lonza Catalog number LT07-318). Morphological assessment was performed using an Olympus CKX41 inverted microscope and by Crystal Violet staining [57]. 

### 2.2. Fluorescence Activated Cell Sorting (FACS) and Flow Cytometry

Cells were harvested with Accutase® (Corning, Catalog # 25-058-CI) and stained with anti-human CD44-FITC (BD Pharmingen), anti-human CD24-PB (Exbio) and anti-human EpCAM-APC (Biolegend) antibodies, as per manufacturer-recommended dilutions for 1 h at room temperature on a rotary shaker. Cells were analyzed in the presence of propidium iodide (1 µg/mL) using a BD LSR Fortessa (BD Biosciences). After doublet discrimination and compensation for spectral overlap, samples were analyzed using FlowJo Software v10.0.7 (BD Biosciences). For sorting, anti-human EpCAM-PerCP/Cy5.5 (Biolegend) antibody was used and cells were sorted using a BD FACS Aria IIu sorter (BD Biosciences).

### 2.3. Single Cell Cloning 

Single cell sorting was carried out in 96-well plates from the whole population as well as after selecting the subpopulations (10%) of cells with the lowest and highest expression of EpCAM respectively, across PMC42-LA on the Astrios flow sorting machine (Beckman Coulter) (Figure 3). The wells were microscopically examined to ensure only single cells were seeded per well across three 96-well plates. Wells were propagated to generate single cell clones in equal proportions of media with PMC42-LA cell-conditioned media. Conditioned media was sourced from 1-week old cultured PMC42-LA cells and was double-filtered prior to its use.

Plates were maintained at 37 °C in a 5% (v/v) CO_2_-humidified atmosphere and were examined every week for the presence of single colonies. After 4 weeks, 36 (12 selected from each 96-well plate) clones were transferred from the 96-well plates into 12-well plates via Passage 1, and then into T25 flasks via Passage 2, and subsequently profiled for EpCAM. The phenotypic stability of four selected clones was monitored throughout the study using EpCAM profiling by flow cytometry. 

### 2.4. RNA Extraction, cDNA Synthesis and Reverse Transcriptase-quantitative PCR (RT-qPCR)

Total RNA was extracted from cells using TRIzol (Life Technologies) and subsequent reactions were carried out as per the Bioline Isolate II RNA Micro kit manufacturer’s instructions. cDNA was synthesized using the SensiFAST^TM^ cDNA Synthesis kit from Bioline. RT-qPCR was performed using the SYBR Green Master Mix in a ViiA7 Real-Time PCR system (Applied Biosystems, Carlsbad, CA, USA) and analysis performed using Quantstudio^TM^ Real-Time PCR software v1.1 (Applied Biosystems, Life Technologies). The primer sequences are listed in Supplementary Table S1.

### 2.5. Western Blotting

Total cell lysates were prepared for each of the EpCAM subpopulations, the four selected PMC42-LA clones, and and parental PMC42-LA cell line by lysing the cells in the presence of RIPA Buffer (10 mM Tris-HCl pH 7.6, 10 mM NaCl, 3mM MgCl2, 1% nonidet P-40, 1 X Protease Inhibitor tablet (Roche)) on ice. Next, protein levels were quantified using the Pierce™ BCA Protein Assay Kit (Sigma) and 30 μg of total protein from each sample was prepared with sample reducing buffer (2 M Urea, 2% SDS (sodium dodecyl sulfate), 0.125 M Tris HCl, 0.1M DTT (dithiothreitol) and bromophenol blue) at a ratio of 3:1 (lysate: reducing buffer) and resolved on an SDS gel with Tris/Glycine/SDS gel running buffer. The samples were subsequently transferred onto nitrocellulose membranes (BioTrace NT, Pall Life Sciences, New York, NY, USA) using a Transblot apparatus (Bio-Rad) and blocked using 1:1 Odyssey® blocking buffer (LI-COR): 1X PBS prior to probing with mouse anti-E-cadherin mAb (clone 36/e-cad, BD Biosciences), mouse anti-vimentin mAb (clone V9, Dako), and mouse Pan-actin mAb (clone ACTN05, Thermo Scientific). Membranes were then scanned on the Odyssey imaging system (Li-Cor, Lincoln, NE, USA) to obtain a visual representation of the amount of protein present in the samples. 

### 2.6. Immunocytochemistry

The EpCAM sorted subpopulation, parental PMC42-LA cells and the single cell-derived clones were seeded at a density of 10,000 cells/well in 48-well plates (Thermo Scientific Nunclon^TM^ Delta Surface-150687). During immunocytochemistry, the growth medium was discarded, and cells were washed thrice gently with Dulbecco’s modified phosphate-buffered saline (DPBS; pH 7.5). Briefly, cells were fixed in 4% paraformaldehyde ± 0.1% Triton X-100 (depending on the desired permeabilization conditions), rinsed with DPBS, and incubated with the designated primary antibodies at 4 °C overnight. After rinsing in DPBS, cells were incubated for 2 h at room temperature in the dark on a gentle rotary shaker with appropriate fluorescence-conjugated secondary antibody (Supplementary Table S2) and with diamidino phenyl indole (DAPI) as a nuclear stain (diluted to a final concentration of 1 µg/mL). The plates were then washed thrice with DPBS and images captured on a high-content imaging platform (Cytell Cell Imaging System (GE Healthcare, Buckinghamshire, UK), IN Cell Analyzer 6000 (GE Healthcare, Buckinghamshire, UK) or PerkinElmer Operetta® (PerkinElmer, Waltham, MA, USA) as indicated) with approximately 9 fields of view taken per well. Images were analyzed and merged using the respective software; IN Cell Investigator software v1.0 (GE Healthcare) or Harmony® v4.8 (PerkinElmer).

### 2.7. Cell Viability Assays

Cells were seeded at 5000 cells/well in a 96-well plate. After overnight incubation, the culture media was changed to include predetermined concentrations of selected drugs (doxorubicin, docetaxel, eribulin) for 72 h. For proliferation rate assessment with and without growth factor EGF, the cells were cultured, and readings were obtained every consecutive 3 days using MTT 3-(4,5-dimethylthiazol-2-yl)-2,5-diphenyl tetrazolium bromide) (Promega) assay. Cell viability for the drug assays was assessed by the resazurin-based Alamar Blue assay (#R7017, Sigma-Aldrich, St. Louis, MO, USA) and the florescence intensity in each well was measured after 1 h using a top-reading florescent plate reader (FLUO Star Omega, BMG LABTECH) with excitation at 544 nm and emission at 590 nm. Untreated cells served as a negative control. The experiments were performed in triplicate.

### 2.8. Incucyte® Migration and Invasion Assay

The cells were seeded in 96-well Essen ImageLock plates (Essen BioScience) to achieve a confluent density (∼5 × 10^5^ per well). After 24 h, cells were treated with mitomycin C (Roche Catalog # 10107409001) for 3 h and scratch wounds were made simultaneously in all culture wells using an Essen WoundMaker. For the Invasion assay using Incucyte, wells were coated with 100 µg/mL basement membrane extract (Cultrex, Trevigen-3433-010-01) in DMEM overnight before cell seeding and, after wound creation, wells were washed to remove dislodged cells and 50 µL of 1 mg/mL of reduced growth factor basement membrane extract diluted in culture mediamedium was added to fill the wound with extra cellular matrix (ECM). The plate was placed in a 37 °C humidified incubator for 1 h to allow the basement membrane to settle, then 50 µL of culture media ±20 ng/mL EGF was added so that the final concentration added was 10 ng/mL. The plates were scanned in the IncuCyte live-cell imaging system (Essen BioScience) at 2-h intervals for 72 h. The data were analyzed with the IncuCyte scratch wound assay software module (Cat No. 9600-0012) and version 2014A.

### 2.9. In Vivo Tumorigenesis

Severe combined immunodeficiency (SCID) mice (eight–ten weeks of age), were purchased from the Animal Resource Centre (ARC, Perth) through the Bio Resources Centre (BRC), St. Vincent’s Hospital, Melbourne, Australia. The in vivo experiments were conducted at the BRC facility. PMC42-LA cells were transduced with the BL2T vector (modified by Dr Bryce van Denderen, St. Vincent Institute (SVI) from L2T clone containing the firefly luciferase 2 and tomato fluorescent gene [58]. The L2T clone was kindly provided by Dr. Michael F. Clarke, Stanford University, CA, USA). Approximately 2 × 10^6^ BL2T PMC42-LA cells were injected into the mammary fat pad of three SCID mice. Nine months post-inoculation, mice were euthanized and the tumors were extracted, mounted with optimal cutting temperature compound (OCT; TissueTek, Sakura Finetek US), snap-frozen in liquid nitrogen-cooled 2-butanol, and stored at −80 °C prior to cryostat sectioning. Before sectioning the tumors onto glass slides, the specimens were processed from OCT to be formalin-fixed, paraffin-embedded. Standard histopathological assessment of the xenografts was performed by haematoxylin and eosin (H&E) staining, and double immunofluorescence staining for EpCAM and vimentin was performed in the Histology core facility at Translational Research Institute, Brisbane, Australia using the BenchMark® ULTRA automated slide stainer (Ventana Medical Systems, Inc., Tucson, AZ, USA). In order to avoid murine stromal contamination in the implanted tumors, all the sections were stained with human-specific V9 mouse monoclonal antibody against vimentin (Roche). 

### 2.10. Preparation of Metaphase Spread 

After 60%–70% cellular confluency was achieved in 60 mm dishes under standard culture conditions, cells were treated with 10 µL of demecolcine (stock: 10 µg/mL) for 3–4 h. Cells were harvested using trypsin (Corning™ 25053CI) and the cell pellet was gently treated with hypotonic solution (75 mM KCl) for 40–60 min at 37 °C and fixed in cold methanol/acetic acid (3:1). Two or three drops of suspended cells were applied to glass slides and chromosomes were stained with DAPI and counted using confocal microscopy (Olympus Fluoview FV1200 Confocal Laser Scanning Microscope, Olympus, Japan).

### 2.11. DNA Extraction, Whole Exome Sequencing and Processing of Sequencing Data 

Genomic DNA was extracted from FACS-sorted EpCAM^High^ and EpCAM^Low^ PMC42-LA subpopulations using the Bioline Isolate II Genomic DNA Kit (Cat: BIO-52067), as per the manufacturer’s instructions. After quantifying the DNA and checking the purity, DNA samples were shipped to GeneWiz, Inc. (Suzhou, China) for whole exome sequencing and subsequent analysis. They performed initial quality control assessments and subsequent exome capture using the SureSelectXT HS Target enrichment kit (Agilent Technologies, Santa Clara, CA, USA). All samples were paired-end multiplex sequenced (2 × 150) on the Illumina Hiseq 2500 platform to a median target depth of over 50×. Paired-end reads underwent quality control before alignment to the reference human genome (hg19) using Burrows-Wheeler alignment (BWA, version 0.7.12-r1039) [59] and SAMtools (version 1.6) [60]. Realignment and recalibration were performed using the Genome Analysis Toolkit (GATK, version 3.5) [61]. Single nucleotide variants (SNVs) and indels were called using GATK with default settings. Annotation of variants (SNP and Indels) was performed using ANNOVAR (http://www.openbioinformatics.org/annovar/) [62]. Control-FREEC v 10.6 was used for detecting and filtering the copy number variations (CNV) [63]. 

### 2.12. Statistical Analysis

All experiments were carried out at least three times unless otherwise indicated. Data were analyzed using GraphPad Prism version 7 statistical software (GraphPad Software, La Jolla, CA, USA).

## 3. Results

### 3.1. EpCAM Expression is Downregulated in Mesenchymal Cells 

**Figure 1:**
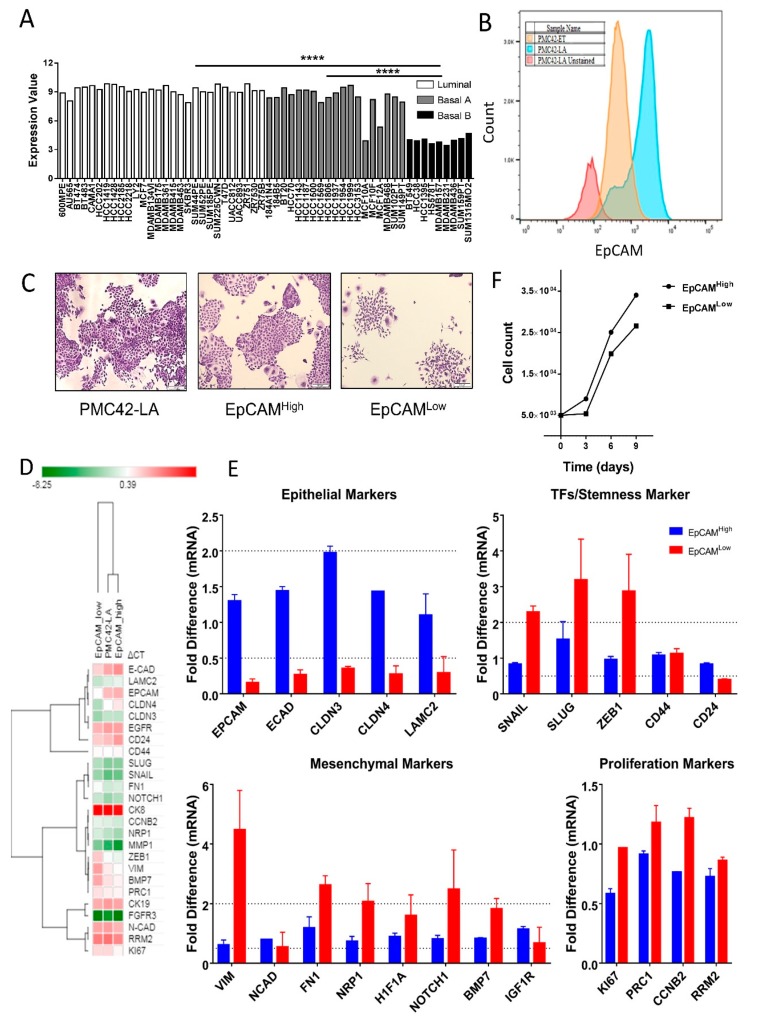
(**A**) EpCAM assessment in gene expression data of 50 breast cancer cell lines and five non-malignant breast cell lines, including three subtypes of luminal, basal A and basal B/mesenchymal. Data are from Array Express (accession no. E-MTAB-181) (Heiser et al., 2012) and are normalized log2-transformed values; **** *P* < 0.0001 (one-way ANOVA, with Tukey’s multiple comparisons). (**B**) Histogram plots depicting differences in the surface levels of EpCAM protein across PMC42-ET and PMC42-LA cell lines. Negative control represents PMC42-LA unstained cells. The EpCAM expression is markedly low in the PMC42-ET parental cell line and the PMC42-LA cell line showed 15%–20% proportion of the population as EpCAM^Low^. (**C**) Crystal violet staining of the colony images of PMC42 LA population and its subpopulations to emphasize the distinct mesenchymal phenotype of the EpCAM^Low^ subpopulation when grown sparsely. (**D**) Hierarchical clustering performed using the Morpheus (Gene-E tool) of the normalized (ΔCt) values. (**E**) Gene expression analysis of 22 genes related to EMT markers and proliferation marker in EpCAM sorted subpopulations relative to expression in the parental (unsorted) PMC42-LA cell line. Data are represented as the mRNA fold difference ± standard error of the mean (SEM) (Results are from *n* = 3 independent biological experiments). (**F**) Proliferation rate for EpCAM^Low^ and EpCAM^High^ subpopulations were evaluated by MTT assay (data are representative of *n* = 3 independent biological experiments).

EpCAM expression as determined by publicly available gene array data is significantly lower in Basal B human breast cancer cell lines, which exhibit enhanced mesenchymal-like features, than in the Luminal and Basal A subgroups (Figure 1A) [64,65]. In the PMC42 system, which clusters with the Basal B cell lines (Eva Tomascovic-Crook, SVI, personal communication), the epithelially shifted PMC42-LA subline has significantly higher expression of EpCAM than the more mesenchymal, parental PMC42-ET cell line (Figure 1B). We found that the PMC42-LA subline comprises an EpCAM^Low^ (mesenchymal) subpopulation in a discrete ratio of 20:80. The presence of an EpCAM^Low^ population suggests an inherent and stable heterogeneity in this subline, which we further characterized on the basis of molecular and phenotypic characteristics and plasticity. EpCAM^High^ and EpCAM^Low^ subpopulations were isolated and analyzed for morphology and expression of epithelial and mesenchymal markers (Figure 1D,E and Supplementary Figure S1D). Crystal violet staining of single cell-seeded, sparsely cultured colonies emphasized their distinct morphology. The EpCAM^Low^ subpopulation cells displayed distinct spindle-like shapes compared to the cobblestone colonies observed in the EpCAM^High^ subpopulation (Figure 1C). Unsupervised hierarchical clustering of the ΔCt values from RT-qPCR of representative epithelial and mesenchymal markers revealed that the EpCAM^High^ population aligned more closely with its parental population, and showed EpCAM^Low^ to be a distinct subpopulation with more mesenchymal features (Figure 1D). The EpCAM^Low^ cells expressed mesenchymal transcripts including vimentin, fibronectin, Notch1 and Neuropilin-1 with concomitant low levels of epithelial transcripts; E-cadherin, claudin-3, claudin-4 and CD24 in EpCAM^Low^ cells were 2-fold lower as compared to parental PMC42-LA cells (Figure 1E). Higher expression of Snail, Slug and Zeb1 was also confirmed in the EpCAM^Low^ subpopulation (Figure 1E). No significant difference was found in mRNA expression of the proliferation marker transcripts despite mesenchymal cell cultures expanding much more slowly than epithelial cells, as shown by the proliferative rate assessment in EpCAM^High^ and EpCAM^Low^ subpopulations, respectively. An initial lag was also observed in the proliferative rate of the EpCAM^Low^ subpopulation (Figure 1F). These results led us to ask further whether isolated epithelial and mesenchymal states proliferate and remain in their purified phenotypic states, or whether the two phenotypes each have the capability to transition back towards the PMC42-LA mixed phenotype.

### 3.2. Cell-State Dynamics in PMC42-LA Breast Cancer Subpopulations 

**Figure 2:**
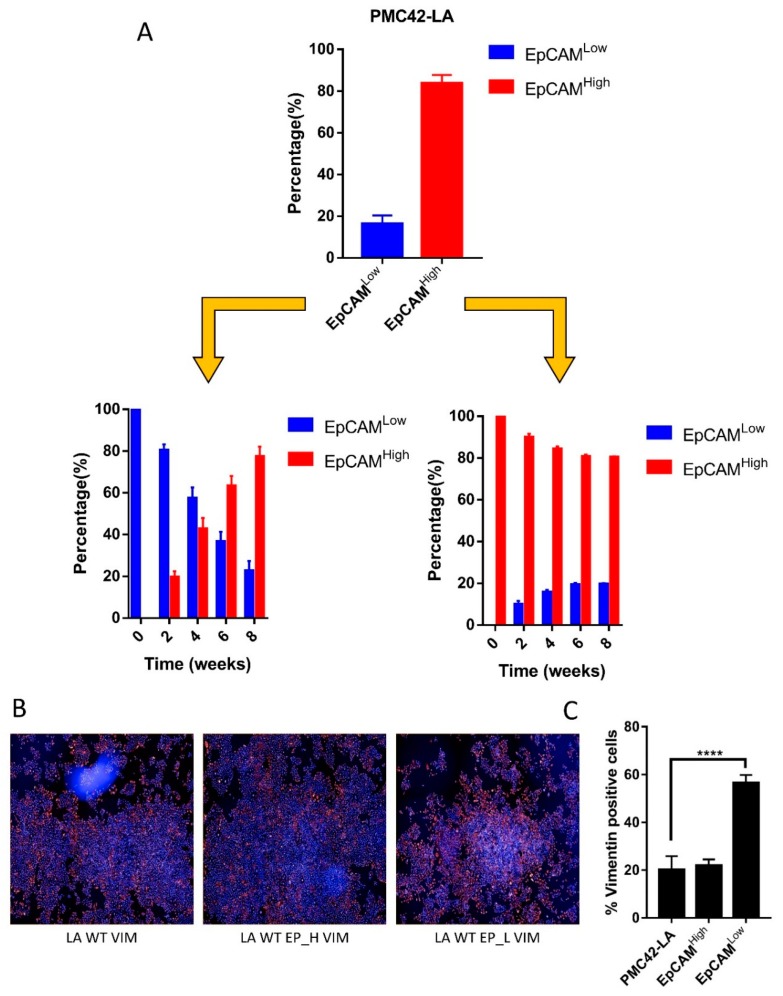
(**A**) Bar charts showing the proportion of cells in EpCAM^low^ and EpCAM^high^ state as intermittently assessed by FACS every two weeks from in vitro culture of FACS isolated EpCAM low and high subpopulations. Data analyzed using repeated measures ANOVA for temporal dynamics signify *P* = 0.0001 for EpCAM high transitions and *P* < 0.0001 for EpCAM Low transitions. (**B**) Immunofluorescence images captured on Operetta high-content imaging system and clustering of nine images at 10× resolution from the center of the well for vimentin expression. (**C**) Bar graph quantifying the number of cells positive for Vimentin expression across PMC42-LA parental and EpCAM sorted subpopulations using Operetta Harmony software. Significant differences were calculated using a paired *t*-test, **** *P* < 0.0001.

**Figure 3:**
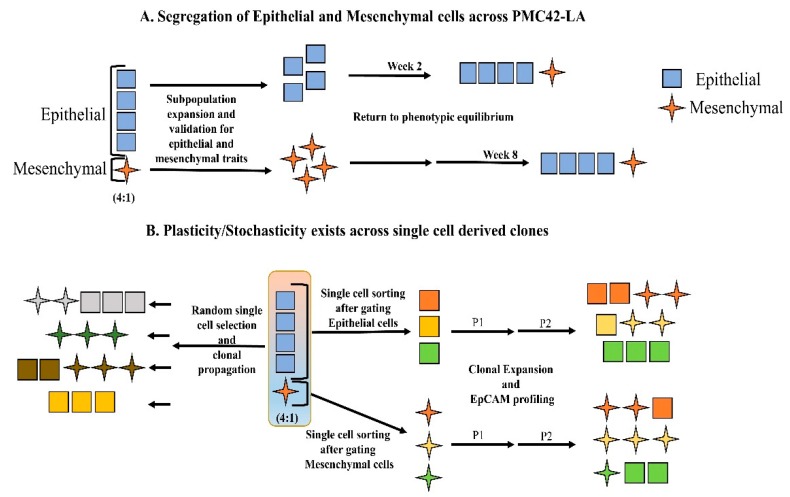
(**A**) Schematic depicting the results of phenotypic equilibrium achieved across sorted and passaged EpCAM subpopulations. (**B**) FACS based single cell sorting and clonal propagation to examine the proportion of epithelial and mesenchymal cells using EpCAM profiling. Single cells were randomly selected across the whole cell population, as well as after gating for epithelial and mesenchymal selection, and seeded in 96-well plates. The progeny of the cells were EpCAM profiled after Passage 2 to identify variation across phenotypic plasticity.

Following isolation of subpopulations of cells that were validated to show distinct epithelial and mesenchymal characteristics, respectively, we sought to determine the potential involvement of EMT and MET in the persistence of these two subpopulations in PMC42-LA cultures. FACS-sorted EpCAM^High^ and EpCAM^Low^ PMC42-LA subpopulations exhibited an average profile of 80:20, respectively. The outlying 10% of the cells in each direction were selected, resulting in subpopulations which were 98%–99% pure, based on post-sort quality control assessment. Sequential EpCAM profiling using FACS was performed every two weeks for eight weeks to evaluate the proportions of epithelial and mesenchymal cells as determined by their EPCAM expression status. For the EpCAM^High^ subpopulation, we observed a rapid progression toward parental equilibrium within two–three weeks. In contrast, the time taken for a return to equilibrium for the EpCAM^Low^ subpopulation was more than eight weeks (Figure 2A). PMC42-LA parental cells and the EpCAM-sorted subpopulations were also imaged for vimentin expression after two passages using immunocytochemistry and high-content imaging, with representative images collated and analyzed using Harmony software (Figure 2B). In the EpCAM^Low^ subpopulation, ~57% of cells were positive for vimentin expression, compared to 18%–21% vimentin-positive cells in both the PMC-42 LA parental and EpCAM^High^ populations (Figure 2C), which validated the results obtained using FACS (Figure 2A). These data revealed that this cell system tends to show a reversion to the parental phenotype transition; hence, single cell sorting and clonal propagation was then performed to gain insight into the dynamics of such inherent cellular plasticity and to investigate the subtleties of this transition beginning from a single cell (Figure 3).

### 3.3. PMC42-LA Tumors Exhibit Small Proportion of EMP 

**Figure 4:**
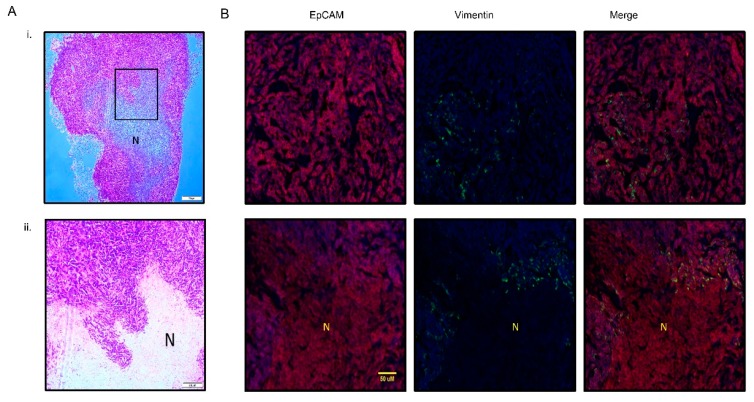
(**A**) Hematoxylin and eosin staining of xenograft PMC42-LA tumor; (*i*) low magnification at 4× (*ii*) high magnification at 10×. (**B**) Representative images (20×) of EpCAM (red), Vimentin (green), and nucleus (blue) staining in PMC42-LA derived tumor from mice. Ubiquitous expression of EpCAM was observed across the cell junctions whereas ~4% vimentin-positive cells were distributed randomly across the whole tumor sectioned slide as well as being present around the necrotic area of tumor. *N*, necrotic area. Scale bar, 50 µM.

We also looked for evidence of plasticity in the PMC42-LA cells in vivo. Standard histopathological assessment of PMC42-LA xenografts was performed initially by H&E staining. The tumor was composed of a large central necrotic area surrounded by viable tissue at the periphery of the tumor (Figure 4A). To assess whether PMC42-LA derived tumors also display a similar proportion of epithelial mesenchymal heterogeneity as found in vitro, a xenograft tumor was immunostained for both EpCAM (red) and vimentin (green) (Figure 4B). Consistent expression of EpCAM was observed across the cell junctions. Overall, quantification from differential staining revealed 3.6% of the cells were vimentin-positive. Vimentin-positive cells (green), which indicate EMT, were clearly seen as clusters in distinct areas of the tumor, specifically at the tumor periphery and at inter-tumoral regions along the tumor-necrosis border. Use of the human-specific V9 anti-vimentin antibody clearly distinguishes the presence of EMT in cancer cells from surrounding mouse stroma.

### 3.4. Generation of Single Cell Clones

#### Phenotypic Plasticity Exists across Single Cell-Derived Clones

**Figure 5:**
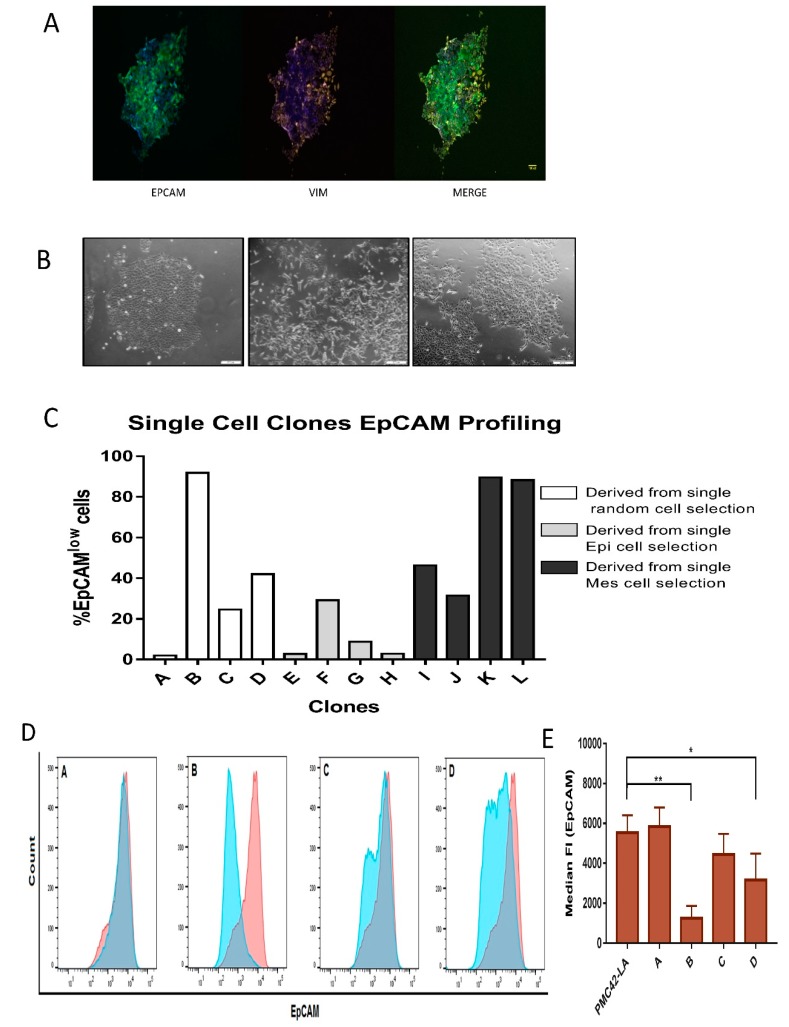
(**A**) EpCAM expression on the cellular junctions and concomitant vimentin-positive cells growing out from an individual cell derived clone (pictures were taken after three weeks of single- cell clonal propagation). (**B**) Morphological assessment of epithelial clustered colony, mesenchymal segregated cells, and mixed epithelial and elongated colonies obtained from clonal propagation of single cells after gating for EpCAM^low^ and EpCAM^high^ cells after the first passage. Scale bar, 200 µM. (**C**) FACS profiling for EpCAM results in distribution of EpCAM low and high cells at variable ratios across various single cell-derived clones. (**D**) Histograms depicting the proportion of EpCAM high and EpCAM low cells in the four selected clones as overlap with parental EpCAM profile (red). (**E**) Staining intensity of EpCAM for the clones and parental PMC42-LA cells assessed by median fluorescence intensity unit (*n* = 4). Significant differences were calculated by one-way ANOVA and nonparametric Dunnett’s multiple comparisons test. * *P* < 0.05, ** *P* < 0.01.

After three weeks in culture, a number of single cell clones were selected, fixed and co-stained for EpCAM and vimentin. Interestingly, in clones derived from the parental PMC42-LA population, differential intrinsic E/M plasticity was observed, with some clones exhibiting spontaneous EMT as evidenced by vimentin staining (Figure 5A). Single-cell clones also demonstrated morphological diversity, with some exhibiting tightly associated cell junctions and tight cobblestone morphology consistent with an epithelial phenotype, while others exhibited spindle-like and elongated features, consistent with a mesenchymal phenotype. Some of the single-cell clones derived from PMC42-LA parental cells also exhibited mixed morphologies, where colonies of both tight clusters and elongated cells could be observed (Figure 5B).

Clones derived from the EpCAM^Low^ subpopulations were validated as having a mesenchymal phenotype when compared to their parental PMC42-LA line, proving the EpCAM profiling by FACS to be a robust method to distinguish and isolate cells along the EMP axis for temporal propagation. Twelve random clones were selected for EpCAM profiling, where 33% of the clones displayed an epithelial phenotype, 25% of the clones displayed a mesenchymal-enriched phenotype, and the remaining 42% of the clones retained a heterogeneous mixture phenotype (Figure 5C). All clones displayed EpCAM profiles that were distinct from the parental population (80:20), highlighting the phenotypic plasticity and stochastic EMP processes that exist in subpopulations of cancer cells.

### 3.5. Characterization of the Four Selected Clones across EMP Axis

**Figure 6:**
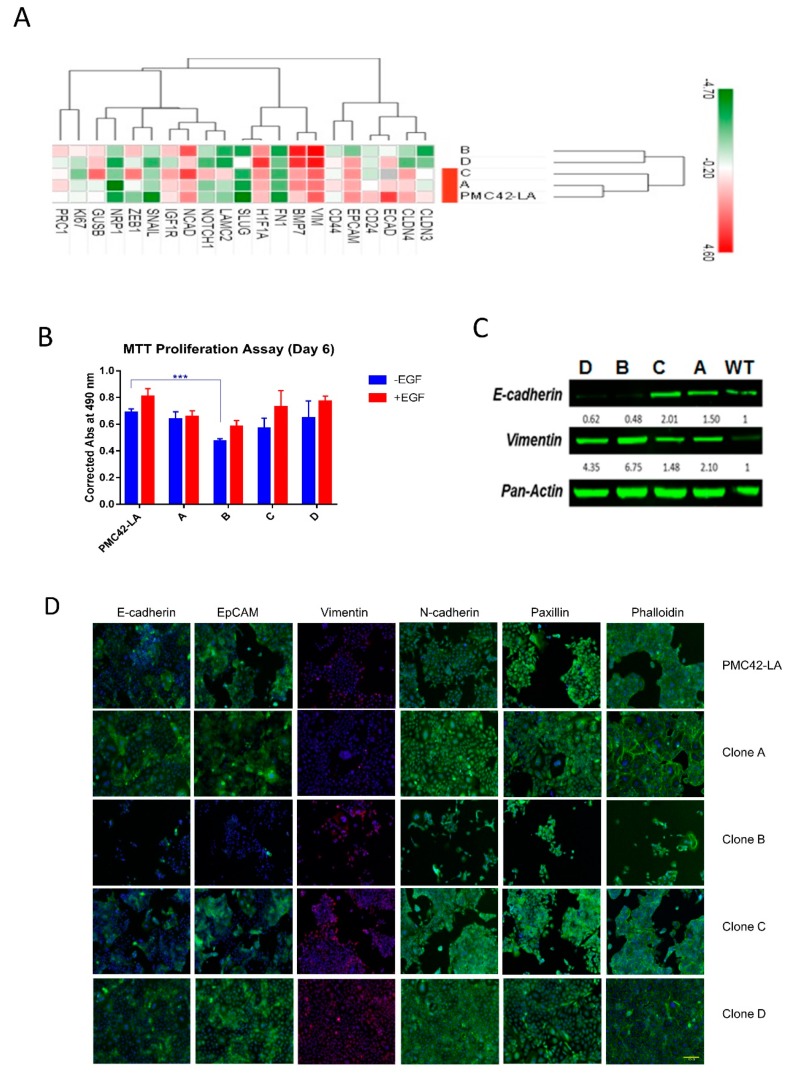
(**A**) Hierarchical clustering of the ΔCt values for the transcriptome data with epithelial-mesenchymal transition (EMT) marker genes for the four clones and PMC42-LA cell line. (**B**) Proliferation rate assessment for the selected clones and parental PMC42-LA with and without epidermal growth factor (EGF) stimulation (*n* = 3). Significant differences were calculated by two-way ANOVA and Sidak’s multiple comparisons test. *** *P* < 0.001 (**C**) The expression of E-cadherin and vimentin as determined by immunoblotting for the clones and parental PMC42-LA cells. Pan Actin was used as the loading control. (**D**) Immunofluorescence microscopy analysis of changes in the localization and expression levels of EMT influencing marker proteins. Selected clones and parental PMC42-LA subline were stained with antibodies against the epithelial markers E-cadherin and EpCAM, against the mesenchymal marker Vimentin and N-cadherin, against paxillin to detect focal adhesion plaques, and with phalloidin to visualize the actin cytoskeleton. Scale bar, 100 μm.

Four clones (Clones A–D) selected according to their differential EpCAM proportions (Figure 5D,E) were further assessed for their intrinsic phenotypes along the EMP axis. Two clones were selected based on predominant EpCAM^High^ (epithelial) and EpCAM^Low^ (mesenchymal) phenotype (Clone A and Clone B), while an additional two were selected due to their mixed nature, containing 75:25 (Clone C) and 60:40 (Clone D) of EpCAM^High^ and EpCAM^Low^ states, respectively. The expression level of 18 EMT marker genes was assessed to score the selected clones according to their EMP status (Figure 6A). Hierarchical clustering for EMP markers reflected the close alignment of Clones A and C with the PMC42-LA parental line, while Clones D and B clustered as a separate clade, exhibiting differential levels of plasticity features at the transcriptomic level with regard to their EMP status. The expression levels of mesenchymal markers were significantly higher for Clone B. Clone C and Clone D display intermediate/mixed phenotypes (consistent with their EpCAM profiling, Figure 5D). PMC42-LA cells are responsive to EGF stimulation for proliferation and EMT induction [57], so clones were also evaluated for the effect of EGF. EGF treatment induced a transcriptionally measurable EMT in Clones A, C and D, but not Clone B, which exhibits a high basal expression of mesenchymal genes, suggesting EGF cannot drive the EMT beyond this point in this system (Supplementary Table S3). Clones were assayed for their proliferation rates and the mesenchymal phenotype Clone B demonstrated significantly lower proliferation rates compared to parental PMC42-LA cells. With EGF stimulation, increases in proliferation were observed for parental and the clonal progenies except for Clone A (Figure 6B).

Immunofluorescence staining revealed a marked difference in the spatial localization and expression of markers for EMP status across the different clones. PMC42-LA and Clone A possessed a predominantly epithelial morphology with segments of EpCAM and E-cadherin expression on the cell junctions, which were missing from Clone B. Each clone, as well as a parental cell line has vimentin-positive cells, however the percentage varies for each clone (Figure 6D). The number and intensity of vimentin-positive cells was higher for Clone B (also supported by Western blot analysis (Figure 6C)) whereas Clone D showed constitutively higher expression of N-cadherin on the cell junctions as compared to parental and other clones. There were subtle differences between the parental PMC42-LA cells and the clones in the cytoskeletal arrangement and focal adhesion formation of the cells clustered in colonies as depicted by phalloidin and paxillin staining, respectively (Figure 6D).

### 3.6. Clone D Demonstrates Enhanced Migratory and Invasive Capacity Compared to Other Clones, but Similar to the Parental Cell Line 

**Figure 7:**
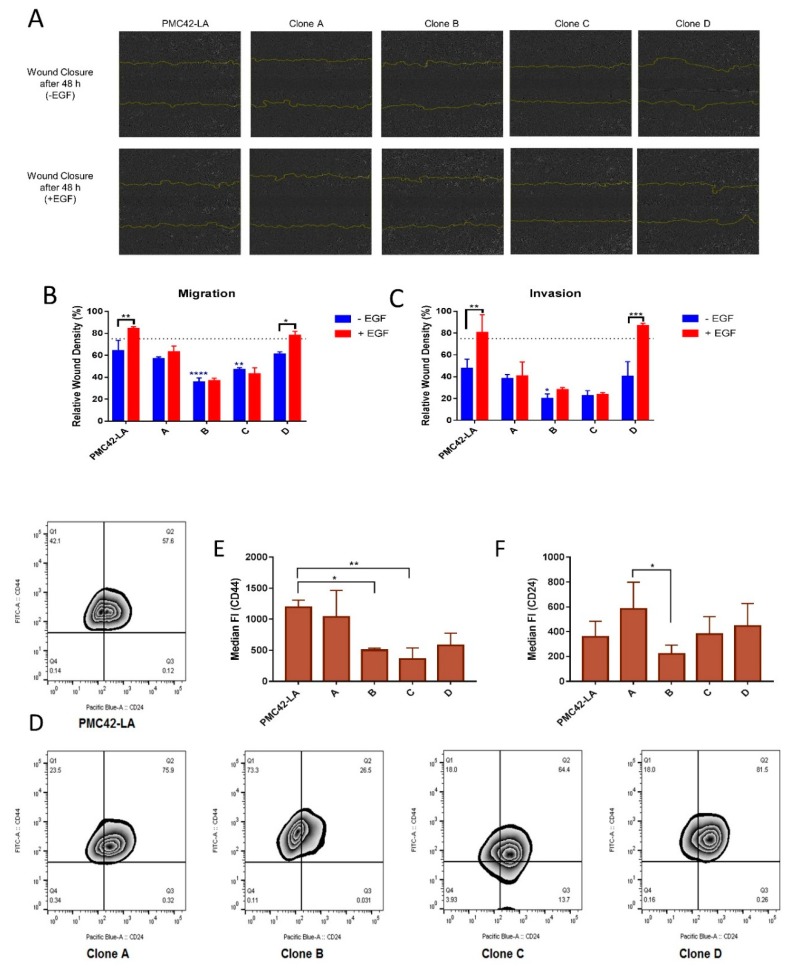
(**A**) In vitro migration capacity of PMC42-LA cells and clone cell lines. The capacity to migrate with and without EGF treatment was measured by live cell imaging in a scratch wound healing assay (IncuCyte ZOOM). Microscope images of migrated PMC42-LA cells and clones are shown after 48 h. Yellow lines denote the original scratch wound. The variation in the density of wound closure with and without EGF treatment is clearly depicted across clones. (**B**) Percentage of relative wound density obtained from IncuCyte™ Scratch Wound Cell Migration. (**C**) Invasion assay after 48 h represented as bar graph. Data are presented as the mean ± std dev of three independent experiments. Significant differences were calculated by two-way ANOVA and Sidak’s multiple comparisons test. * *P* < 0.05, ** *P* < 0.01, *** *P* < 0.001, **** *P* < 0.0001 (**D**) Zebra plot showing the flow cytometry surface staining of CD44 and CD24 expression markers on parental and clonal progenies of PMC42-LA. (**E**) Staining intensity of CD44 assessed by median fluorescence intensity unit (*n* = 4). (**F**) Staining intensity of CD24 assessed by median fluorescence intensity unit (*n* = 4). Significant differences were calculated by one-way ANOVA and nonparametric Dunnett’s multiple comparisons test. * *P* < 0.05, ** *P* < 0.01.

Analyzing collective cell migration in a scratch wound assay, we found that Clone D migrated comparably to the parental cell line, whereas Clones B and C were significantly slower to repair the wound. Only the parental PMC42-LA cells and Clone D showed an increased rate of wound closure with EGF treatment relative to their unstimulated counterparts (Figure 7A,B). At the end time point of the assay, i.e., after three days, cells were fixed and stained with vimentin antibody. Vimentin-positive cells were observed along the wound edge of all the clones and the parental PMC42-LA cells (Supplementary Figure S2A). Interestingly, Clone B with high endogenous vimentin expression did not possess a strong migratory phenotype in this assay. Using Matrigel to mimic invasion through the basement membrane and into ECM, the parental cell line PMC42-LA and Clone D displayed the strongest invasive phenotype, and only clone D and the parental PMC42-LA cells were more invasive after EGF stimulation compared to untreated cells. The invasive capacity was thus similar for the clones and parental cell line compared to that of the migratory phenotype, despite a drastic reduction in the extent of wound closure after 72 h (reduced by ~20% in the absence of EGF) (Figure 7C). 

### 3.7. Variation in Stemness Traits across the Clones and PMC42-LA

Next, the parental cell line and its derivative clone were assessed for their stemness properties using CD44 and CD24 markers. Interestingly, a biphasic population distribution for CD24 expression was observable for the PMC42 parental line but not in any of the sub-clones. The median fluorescence intensity of CD44 was lower for Clones B, C and D compared to the parental line and Clone A (Figure 7D). Low CD24 expression also correlated positively with lower EpCAM expression in Basal B cell lines (Supplementary Figure S1C) [65]. Clone B, with the lowest EpCAM expression showed 73.3% of cells within the CD24 low fraction, whereas the remaining clones possessed a CD24 low fraction (Q1: representing CD44 high, CD24 low) of less than 25% (Figure 7D). The EpCAM^Low^ subpopulation also had a marked increase (~10%) in their CD44^High^/CD24^Low^ “stem-like” population relative to the parental cell line (Supplementary Figure S1A,B), and is consistent with RT-qPCR results showing consistent CD44 expression but 2-fold downregulation in CD24 expression compared to parental PMC42-LA cells (Figure 1E). 

### 3.8. Variable Drug Resistance of Single Cell-Derived Clones of PMC42-LA

**Figure 8:**
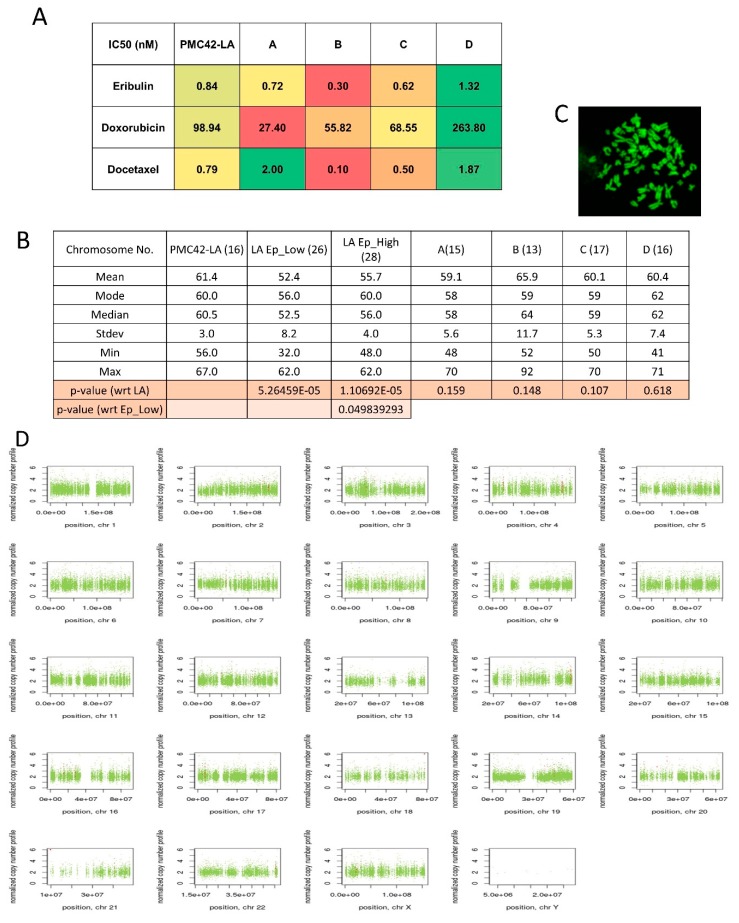
(**A**) Plot of heatmap on the basis of computed IC50 values of the drugs eribulin, doxorubicin, and docetaxel for PMC42-LA and the four selected clones. (**B**) Measurements of central tendency from distribution of chromosome number across PMC42 LA, EpCAM sorted subpopulations and four clones. Student *t*-test was applied to calculate *p*-value. (**C**) Metaphase spreads of PMC42-LA chromosomes stained with DAPI and imaged with confocal microscopy. (**D**) Visualization of Control-FREEC v6.0 output from PMC42-LA sorted EpCAM subpopulations whole exome sequencing data (Illumina HiSeq 2000). Copy number profiles for all chromosomes are shown for EpCAM^low^ subpopulation in comparison to EpCAM^high^; normal copy number status is shown in green, copy number gains are represented in red, and copy number losses are represented in blue.

The chemotherapeutic sensitivity of PMC42-LA and sub-clones was also investigated with doxorubicin, eribulin and docetaxel. The half maximal inhibitory concentration (IC50) of parental PMC42-LA and the selected clones was determined using serial 3-fold dilutions of each drug, followed by Alamar Blue assay. The IC50 of parental PMC42-LA was calculated as 98.94 nM for doxorubicin, 0.83 nM for eribulin and 0.79 nM for docetaxel (Supplementary Figure S2). The sub-clones showed variable response to the different chemo-treatments (Figure 8A). This assay revealed that Clone D was significantly more resistant than the other clones and the parental cell line across all three drug treatments. These data demonstrate that in this cell system, the epithelial or mesenchymal enriched sub-clones were surpassed by Clone D (with mixed phenotype states of 60 epithelial: 40 mesenchymal cells) in their chemo-resistance phenotype. 

### 3.9. Chromosomal Instability (CIN) Reflected across EpCAM-Sorted Subpopulations 

In order to determine the extent to which CIN may be associated with the intrinsic plasticity of PMC42-LA cells, we performed metaphase spreads and counted the abnormal chromosome numbers from parental PMC42-LA cells, EpCAM^Low^ and ^High^ subpopulations, and the four single cell-derived clones from PMC42-LA. The EpCAM^Low^ and EpCAM^High^ subpopulations showed a significant deviation in their chromosome ploidy distribution, whereas numerical chromosomal aberrations per clone did not differ significantly from those of parental cell line PMC42-LA (Figure 8B,C). 

In order to deeply examine the influence of chromosomal instability, whole exome sequencing (WES) of the sorted EpCAM^Low^ and EpCAM^High^ subpopulations and PMC42-LA cells was undertaken; however, comparing the copy number variation (CNV) data deciphered via WES for the EpCAM^Low^ subpopulation to the EpCAM^High^ subpopulation did not reveal any significant differences in the ploidy (Figure 8D). The data analyzed showed that the EpCAM^Low^ and EpCAM^High^ sorted subpopulations were not very different genetically. 

## 4. Discussion

### 4.1. Dynamic EMT and MET Changes Observed in EpCAM-Profiled Subpopulations 

Our findings established that epithelial and mesenchymal subpopulations, defined by their EpCAM expression, exist within the PMC42-LA breast cancer cell line, which maintains on average an EpCAM^+/High^ epithelial and EpCAM^Low^ mesenchymal population ratio of 80:20; whereas, the panel of other luminal cell lines (MCF7, T47D) and basal cell lines (MDA-MB-231, Sum159, HCC38), FACS profiled using EpCAM, displayed a uniform distribution of EpCAM high and EpCAM low states. Bidirectional transitions observed between the sorted epithelial and mesenchymal subpopulations in PMC42-LA suggest that intercellular regulation may exist to direct a phenotypic equilibrium inherent to the parental cell line. The time taken to achieve such a stable equilibrium from the purified mesenchymal subpopulation was longer than eight weeks and contrasts with studies with the SUM159 and SUM149 cell lines, where a phenotypically stable equilibrium was observed to occur rapidly after six days of growth [29]. CD44^low^ non-CSC populations, isolated from five different basal breast cancer cell lines, also reported a return to CD44^high^ state in vivo [66]. The dynamic EMT and MET was also observed in parental and HCC38 cells delineated by EpCAM profiling [26], in Zeb1 driving CD44^Low^ to CD44^High^ cellular plasticity [58] and in mammary carcinoma mouse MyPT models delineated by E-cadherin profiling [30]. Autocrine signaling is also speculated to play a significant role in EMP dynamics [67]. Recently, the exhibition of hysteretic patterns in TGF-β driven EMT also illustrated bi-stability of cellular states in tumor mammary epithelial cells, related to a higher propensity for metastatic colonization [68]. 

### 4.2. Inherent Phenotypic Plasticity and Differential Functional Attributes of the Single Cell-Derived Clones

The inherent plasticity was also evaluated in the sub-clones after isolating the single cells by their epithelial and mesenchymal traits as determined by their relative EpCAM high or low states. The proportion of epithelial and mesenchymal states varied across as well as within the sub-clones (Figure 3B and Figure 5C) and also illustrates/renders the possibility of bi-directional phenotypic transitioning (interconversion between epithelial and mesenchymal states). None of the clones profiled for EpCAM displayed a similar distribution of EpCAM high and low states (80:20) as present in the parental PMC42-LA line, suggesting that stochastic fluctuations and inter-clonal cooperativity creates a special equilibrium [69,70], which can be of extreme relevance in mediating metastasis [71]. This plasticity across the EMP spectrum also elicits variable cellular behaviors, which may impact their tumorigenicity, therapy resistance, and proliferation. 

The distinct clonal progenies derived from the parental line PMC42-LA displayed marked phenotypic heterogeneity. The presence of sub-clonal variants that exhibit phenotypic diversity across the epithelial-mesenchymal axis from populations of single cells in prostate and breast cancer, has also been verified recently, from in-vitro settings [42,46,72]. Observations based on assessing the four clonal populations in this study for their proliferation, transcriptional EMP status, migration, invasion, stemness, and chemoresistance demonstrated dynamics of intra-tumoral variability in the clones at a functional level. The presence of vimentin proved that the cells on the wound edge exhibited enhanced EMT consistent with cellular movement. However, the lower migratory phenotype in the mesenchymal Clone B led us to suspect that this context may require crosstalk as well as additional stimulation, or possess a defect in the way polarity proteins and extracellular proteins required for movement are trafficked [73,74]. The apparent differences observed in the expression and localization of various mesenchymal markers, such as marked increased in N-cadherin expression on the cell junctions in the Clone D also suggest multifactorial regulatory circuits, not only at the RNA or epigenetic level, but also at the protein level, that can impact intratumoral heterogeneity [75]. The level of CD24 was ~2-fold lower in the EpCAM^Low^ population compared to the parental PMC42-LA cells, and was thus enriched for stem cell-like properties through enhancing their CD44(high)/CD24(low) ratio. Low CD24 expression also correlated positively with lower EpCAM expression in Basal B cell lines (Supplementary Figure S1C) [65]. The marked differences apparent in sub-clones for their proportion of CD44^High^/CD24^Low^ cells also highlight the additional clonal diversity at stemness level, and its relation to tumorigenic potential warrants further investigation. The differential expression of stemness markers was also consistent with the stochastic behavior of the sub-clones, in their response to next evaluated chemo-sensitivity. 

Interestingly, in our PMC42-LA single cell-derived clones, the relatively slow proliferating clone with enriched mesenchymal traits (Clone B) did not possess high chemo-resistance against the panel of drugs tested, while counterintuitively, Clone D (with mixed phenotype states of 60 epithelial:40 mesenchymal) had more survival benefit as compared to the parental line and other clones. These results are in line with similar observations found in single cell-derived prostate cancer clones, where mesenchymal features (capable of undergoing EMT) did not necessarily enhance therapy resistance [72]. 

### 4.3. Chromosomal Instability Doesn’t Attribute to Intrinsic Phenotypic Plasticity 

We also observed that copy number variations from whole exome sequencing of EpCAM low versus high subpopulations did not correlate with significant differences seen at the chromosome level in ploidy analysis of metaphase spreads between the epithelial and mesenchymal subpopulation in comparison to PMC42-LA. Very few changes were seen at the somatic mutation or CNV level, and further validation from WES studies may be warranted. As presented in Supplementary Tables S4 and S5, several microRNAs (e.g., MIR-3648, MIR-3687) were highly amplified in copy number in the EpCAM^Low^ subpopulation. These results indicate that intrinsic plasticity is contributed by factors other than CIN. Studies examining the contribution of genetic mutations to phenotypic plasticity within tumors and cell lines have resulted in inconsistent conclusions [76,77,78,79]. Determinants of metastatic competency investigated by sequencing of primary tumors and metastases from various cancers, such as colorectal cancer and ovarian cancer, have been unable to link specific genetic alterations with tumor dissemination per se [42,80]. Intra-tumoral heterogeneity beyond genetic determinants also had clinical implications in chemotherapy response [81,82,83]. Both intra-tumoral heterogeneity and intrinsic cellular plasticity warrant consideration as important non-genomic factors that may contribute to dynamic cellular behaviors. Various factors at the cellular or sub-cellular level, such as oscillations of gene expression by epigenetics, alternate splicing, or other unknown factors can also propagate cancer progression [84]. Most recently, the contribution of conformational dynamics of intrinsically disordered proteins, such as oncoproteins, reprogramming transcription factors (TFs) and EMT-TFs in cancer cells was also recognized [85,86,87,88]; these can also endow the cells with phenotypic diversity and robust survival potential during chemotherapy regimens. The computational models have also provided a rationale in decoding these intrinsic dynamics/the cell state transition of EMT based on epigenetic regulation and gene regulatory networks [89,90,91,92]. Further, the identification of tumor transition states occurring during EMT via phenotypic markers [25] and using a theoretical experimental framework approach to determine the plastic interplay of cell phenotypes [93] can herald a major refinement of our understanding of the intra-tumoral heterogeneity and plasticity within the tumor. 

This work provides insight into the paradigm of the dynamic heterogeneity that exists within cancer cell populations and defines the contribution of intrinsic plasticity that endows the functional and phenotypic diversity to allow cancers to adapt within the tumor environment. It thus becomes imperative to develop approaches that allow us to estimate and model these dynamic processes that drive intra-tumoral heterogeneity and cellular plasticity. Tailored approaches need to be developed in such a way that therapy should not only reduce the tumor burden and prevent metastasis, but also address intra-tumoral heterogeneity to prevent adaptive responses.

## Supplementary Materials

The following are available online at https://www.mdpi.com/2077-0383/8/6/893/s1, Figure S1: (A) FACS analysis of cell surface markers CD44 and CD24 in parental and EpCAM sorted low and high cells of PMC42-LA. (B) Percentages of the CD44^+^CD24^Low^ cells assessed through FACS in PMC42-LA and EpCAM sorted low and high subpopulations. (C) CD24 assessment in gene expression data of 50 breast cancer cell lines and 5 non-malignant breast cell lines, including three subtypes of luminal, basal A and basal B/mesenchymal. Data are from Array Express (accession no. E-MTAB-181) (Heiser et al., 2012) and are normalized log2-transformed values; * *P* < 0.05, ** *P* < 0.01 (one-way ANOVA, with Tukey’s multiple comparisons). (D) Immunofluorescence microscopy analysis of EpCAM high and low sorted subpopulations of PMC42-LA cells using Cytell. Cells were stained with antibodies against E-cadherin, Vimentin, and EpCAM. (Scale bar, 100 µM), Figure S2: (A) Immunofluorescent staining for vimentin (green) reveals all cells in the vicinity of wound closure are vimentin positive for parental cell line and the clones. Scale bar: 100 μM. (B) Growth inhibitory effect of Eribulin, Doxorubicin, Docetaxel in PMC42-LA cell line after 72 h exposure. Table S1: List of Primers used in RT-qPCR annotated with their Gene Symbols and forward and reverse primer sequences information, Table S2: Antibodies used in this study along with their clone, supplier and catalog number information, Table S3: Fold change regulation of various EMT markers in PMC42-LA and single-cell derived clones after 10 ng/ml of EGF treatment for 3 days, Table S4: Copy Number Variations reflected in EPCAM_High vs. EpCAM_Low subpopulation of PMC42-LA cells, Table S5: SNVs identified in EpCAM_Low subpopulation wrt EpCAM_high subpopulation of PMC42-LA cells.
