# Supplementary material for: Interrogation of Phenotypic Plasticity between Epithelial and Mesenchymal States in Breast Cancer
Source: J Clin Med. 2019 Jun 21;8(6):893. doi: 10.3390/jcm8060893 (PMC6617164; doi:10.3390/jcm8060893)
Supplement: Supplementary file 1 [file jcm-08-00893-s001.zip › jcm-525570-supplementary figures.docx]

**
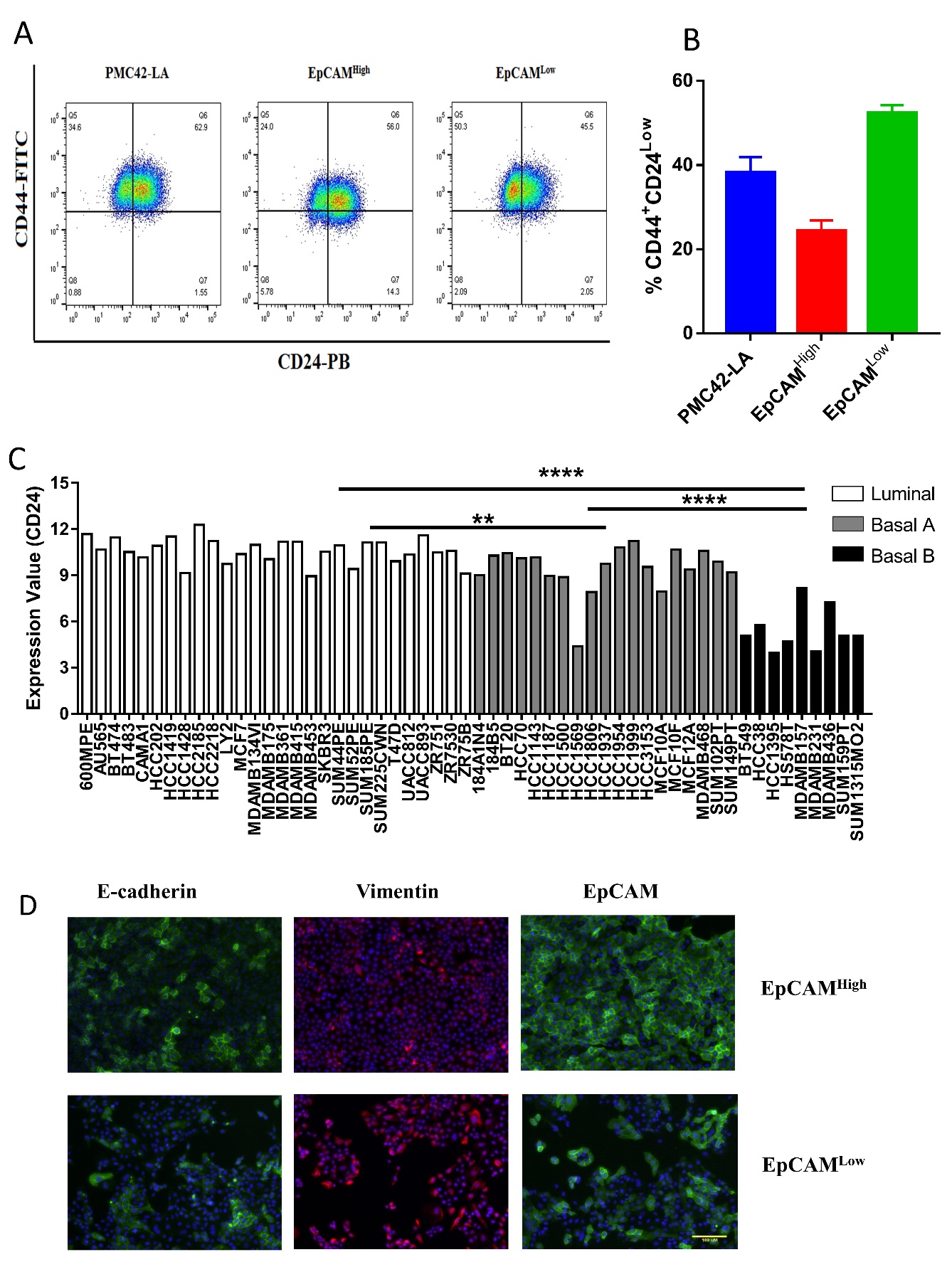
**

**Supplementary Figure 1: (A) FACS analysis of cell surface markers CD44 and CD24 in parental and EpCAM sorted low and high cells of PMC42-LA.** (**B**) Percentages of the CD44^+^CD24^Low^ cells assessed through FACS in PMC42-LA and EpCAM sorted low and high subpopulations. (**C**) CD24 assessment in gene expression data of 50 breast cancer cell lines and 5 non-malignant breast cell lines, including three subtypes of luminal, basal A and basal B/mesenchymal. Data are from Array Express (accession no. E-MTAB-181) (Heiser et al., 2012) and are normalized log2-transformed values; * *P* < 0.05, ** *P* < 0.01 (one-way ANOVA, with Tukey’s multiple comparisons). (**D**) Immunofluorescence microscopy analysis of EpCAM high and low sorted subpopulations of PMC42-LA cells using Cytell. Cells were stained with antibodies against E-cadherin, Vimentin, and EpCAM. (Scale bar, 100 µM).


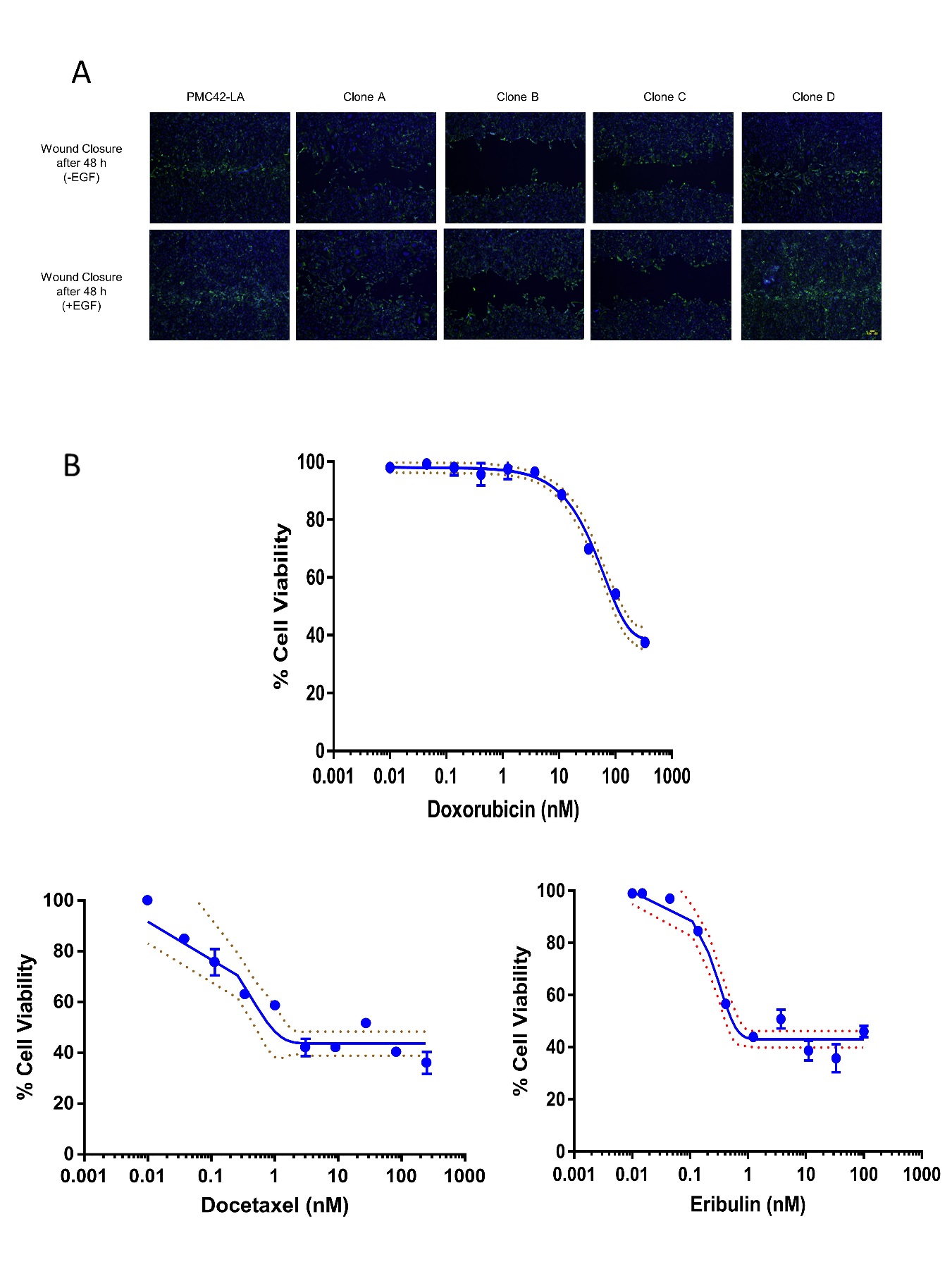


**Supplementary Figure 2:** **(A)** Immunofluorescent staining for vimentin (green) reveals all cells in the vicinity of wound closure are vimentin positive for parental cell line and the clones. Scale bar: 100 μM. (**B**) Growth inhibitory effect of Eribulin, Doxorubicin, Docetaxel in PMC42-LA cell line after 72 h exposure.
